# Blood Pressure Reduction Effects of Salt Substitutes in Different Application Settings A Meta-Analysis of Randomized Controlled Trials

**DOI:** 10.1016/j.jacasi.2026.02.014

**Published:** 2026-04-01

**Authors:** Xianghui Zhang, Yifang Yuan, Chao Gao, Xuejun Yin, Yufei Ji, Xizi Zheng, Qi Zhou, Yangfeng Wu

**Affiliations:** a Department of Epidemiology and Biostatistics, School of Public Health, Shihezi University, Shihezi, China; b Clinical Research Institute, Institute of Advanced Clinical Medicine, Peking University, Beijing, China; c National Institute for Nutrition and Health, Chinese Center for Disease Control and Prevention, Beijing, China; d School of Public Health, Jiangxi Medical College, Nanchang University, Nanchang, China; e Fuwai Hospital, State Key Laboratory of Cardiovascular Disease, National Center for Cardiovascular Diseases, Peking Union Medical College, Chinese Academy of Medical Sciences, Beijing, China; f Renal Division, Peking University First Hospital, Institute of Nephrology, Peking University, Beijing, China; g Evidence-Based Medicine Center, School of Basic Medical Sciences, Lanzhou University, Lanzhou, China; h Department of Epidemiology and Biostatistics, School of Public Health, Peking University, Beijing, China; i State Key Laboratory of Vascular Homeostasis and Remodeling, Peking University, Beijing, China

**Keywords:** application setting, blood pressure, meta-analysis, salt substitutes

## Abstract

**Background:**

Meta-analysis has been performed on the blood pressure–lowering effect of salt substitutes; however, whether the effects vary by application setting remains unclear.

**Objectives:**

This study aims to compare the blood pressure–lowering effect of salt substitution between home-cooking and collective-cooking settings.

**Methods:**

We searched PubMed, EMBASE, the Cochrane Library, CNKI, and WanFang databases for randomized controlled trials (RCTs) comparing salt substitutes with usual salt on blood pressure, with the last search on June 3-4, 2025. Meta-analysis stratified by application setting was performed.

**Results:**

Participants (N = 29,570) in 21 RCTs (n = 27,813 participants in 18 RCTs with home-cooking and n = 1,757 participants in 3 RCTs with collective-cooking) were included for analysis. Salt substitutes significantly reduced systolic blood pressure (SBP) by 4.7 mm Hg (95% CI: –5.7 to –3.6; I^2^ = 65.3%) and diastolic blood pressure (DBP) by 1.4 mm Hg (95% CI: –1.7 to –1.0; I^2^ = 17.3%) compared to usual salt. The effect varied by application setting: in home-cooking, SBP and DBP were reduced by 4.2 mm Hg (95% CI: –5.3 to –3.2; I^2^ = 61.5%) and 1.2 mm Hg (95% CI: –1.6 to –0.9; I^2^ = 14.4%), respectively; in collective-cooking, reductions were substantially larger — 7.7 mm Hg (95% CI: –10.0 to –5.3; I^2^ = 0%) and 2.4 mm Hg (95% CI: –3.8 to –1.1; I^2^ = 0%), respectively. The between-setting difference was significant for SBP (*P* = 0.008) and borderline significant for DBP (*P* = 0.099). Sensitivity analyses restricted to high-quality studies or other study subsets, as well as meta-regression adjusting for multiple variables, generally supported these findings.

**Conclusions:**

Replacing usual salt with salt substitutes lowered blood pressure more in the collective-cooking than the home-cooking setting, suggesting that public health policies and strategies should be developed to prioritize full adoption of salt substitutes to maximize its health benefits.

Elevated blood pressure is the leading risk factor of cardiovascular disease, accounting for approximately two-thirds of stroke and nearly half of coronary heart disease.^1^^,^^2^ Both excessive sodium intake and insufficient potassium intake contribute to blood pressure rise and hypertension development, thereby increasing the risk of cardiovascular disease morbidity and mortality.^3^, ^4^, ^5^, ^6^ The World Health Organization (WHO) has recommended that both adults and children reduce sodium intake and increase potassium intake.^7^^,^^8^ However, the most recent studies have shown that the global mean intakes of sodium and potassium remained far from the WHO recommended targets.^9^

Potassium-enriched salt substitutes, which replace a proportion of sodium chloride with potassium chloride, simultaneously reduce sodium intake and increase potassium intake, providing a novel strategy for sodium reduction and potassium supplementation. A previous systematic review of randomized controlled trials (RCTs) demonstrated that replacing usual salt with salt substitutes significantly reduced systolic blood pressure (SBP) and diastolic blood pressure (DBP) on average by 4.6 mm Hg and 1.6 mm Hg, respectively.^10^ More recently, the DECIDE-Salt (The Diet, Exercise and Cardiovascular Health–Salt) study—a large-scale cluster RCT conducted among residents of care homes—implemented salt substitutes in a collective-cooking setting, resulting in an even greater reduction in blood pressure, with an SBP mean decrease of 7.1 mm Hg and a DBP mean decrease of 1.9 mm Hg.^11^

The findings from the DECIDE-Salt study suggest that the blood pressure–lowering effect of salt substitutes may differ by the application settings. Therefore, we hypothesized that replacing usual salt with salt substitutes in a collective-cooking setting would lead to greater blood pressure reductions than in the home-cooking setting. To test this hypothesis, we systematically searched for all RCTs worldwide that compared salt substitutes with regular salt for lowering blood pressure and conducted a meta-analysis stratified by the cooking settings in which salt substitutes were applied.

## Methods

### Study selection

We conducted a comprehensive literature search for English-language articles in PubMed, Embase, and the Cochrane CENTRAL, as well as for Chinese-language articles in CNKI and WanFang databases. The last search was conducted on June 3, 2025, for Embase and the Cochrane CENTRAL and on June 4, 2025, for PubMed, CNKI, and WanFang. We included RCTs that reported the effect of salt substitutes on blood pressure; in these studies, the substitutes contain at least 10% potassium chloride. We excluded protocols, reviews or commentaries, trial registrations, studies published only as conference abstracts, and trials without a usual salt control group. The search strategy incorporated keywords such as “salt substitute,” “low sodium salt,” “blood pressure,” “systolic blood pressure,” and “diastolic blood pressure,” and their corresponding terms in Chinese. The detailed search strategy is shown in the Supplemental Material. The present study is an extension derived from the evidence review conducted for The Chinese Guidelines on Use and Promotion of Low Sodium Salt (PREPARE-2023CN660).

### Data extraction

A standardized data extraction form was used to collect information on study characteristics, baseline characteristics of the study population, and outcomes. Study characteristics included author, year of publication, country, sample size, application setting of the salt substitute, proportion of potassium chloride in the salt substitute, and intervention duration. Baseline characteristics comprised mean age, proportion of men participants, and mean blood pressure. The outcomes were SBP and DBP. The intervention effect estimates on SBP and DBP were extracted for the primary analysis results as reported in each original trial, no matter if covariables were adjusted for or not. Effect estimates were extracted following a prespecified hierarchy, consistent with the Cochrane Handbook for Systematic Reviews of Interventions.^12^ First, when trials directly reported the mean difference in change (difference in differences, DID) with corresponding variance, these values were extracted unchanged. Second, when changes from baseline were reported but the between-group difference was not, the DID was calculated from the available data, and variances were derived using group-specific standard deviations (or standard errors) of change values and sample sizes. Third, when neither the DID nor change-from-baseline values were available, mean and SDs of blood pressure at both baseline and end of intervention, along with sample sizes, were extracted to reconstruct the DID and its variance for data synthesis. All 3 cluster RCTs reported appropriately cluster-adjusted effect estimates; therefore, no further adjustment was applied when incorporating these data into the pooled analyses.

In our study, the home-cooking setting referred to settings in which salt substitutes were provided to free-living participants to replace usual salt in home cooking and food preparation, with actual use fully dependent on participants’ compliance with study instructions. In contrast, the collective-cooking setting referred to settings in which salt substitutes were used to replace usual salt for the cooking, preparation, or processing of foods that were centrally provided to participants — either through institutional kitchens where participants lived collectively or by study staff in a feeding-trial setting. In these collective-cooking settings, participants had little or no autonomy over the amount of salt substitute used in their meals. The application setting was independently classified by 2 authors based on the methods section of each trial, and the rationale for each classification was documented (Supplemental Table 1).

### Quality assessment

The revised Cochrane risk of bias assessment tool for randomized trials (ROB2)^13^ was used to assess the risk of bias for each included RCT on the following domains: 1) randomization process; 2) deviations from intended interventions; 3) missing outcome data; 4) measurement of the outcome; and 5) selection of the reported result. Two authors (X.Z. and Y.Y.) independently performed study selection, data extraction, and risk of bias assessment. Any discrepancies were resolved through discussion until consensus was reached.

The GRADE (Grading of Recommendations Assessment, Development and Evaluation) approach^14^ was used to assess the overall certainty of evidence for the pooled estimated effects of salt substitutes on SBP and DBP; results of this process initiated with high certainty but were downgraded based on risks of bias, inconsistency, indirectness, imprecision, and publication bias.

### Ethical approval

Institutional review board approval was not required for this study because it is a meta-analysis based solely on data from published literature.

### Statistical analysis

The effect of salt substitutes on blood pressure was estimated as the mean difference (MD) in change from baseline between the salt substitute and usual salt groups, with corresponding 95% CIs and 95% prediction intervals using a random-effects model based on a priori hypotheses that the effect may vary by application settings. Subgroup meta-analysis was performed with stratification of application settings. The random-effects model was used because the effects among studies may vary by application setting, baseline blood pressure, proportion of potassium chloride in salt substitutes, and duration of intervention, et cetera. The DerSimonian–Laird random-effects model was selected to facilitate comparability with previous meta-analysis on salt substitutes.^15^ Statistical heterogeneity across studies was assessed using Cochran’s Q test and quantified with the I^2^ statistic.

To assess the robustness of our findings, we performed several sensitivity analyses: 1) restricting the analysis to RCTs with low risk of bias; 2) estimating with a multivariate meta-regression analysis adjusting for baseline mean blood pressure, mean age (≥0 years vs <60 years), proportion of potassium chloride in salt substitutes, region (outside China vs China), intervention duration (≥12 months vs <12 months), and risk of bias; 3) restricting to individual RCTs; 4) restricting to RCTs conducted outside China; 5) restricting to RCTs using salt substitutes containing ≤30% potassium chloride; and 6) restricting to RCTs with a mean participants age ≥60 years. In addition, to further evaluate the robustness of our results, a leave-one-out sensitivity analysis was conducted by sequentially excluding one study at a time. For each iteration, subgroup meta-analysis with stratification by application setting was conducted using the same random-effects model to recalculate the pooled MDs and corresponding 95% CIs to assess whether the observed between-setting differences were driven by any single study.

The characteristics of the included RCTs were presented in tables, and results from primary and sensitivity analyses were presented using forest plots. All analyses were conducted using R software, version 4.2.2 (R Project for Statistical Computing). A 2-sided *P* < 0.05 was considered statistically significant.

## Results

### Characteristics of included RCTs

**Figure 1:**
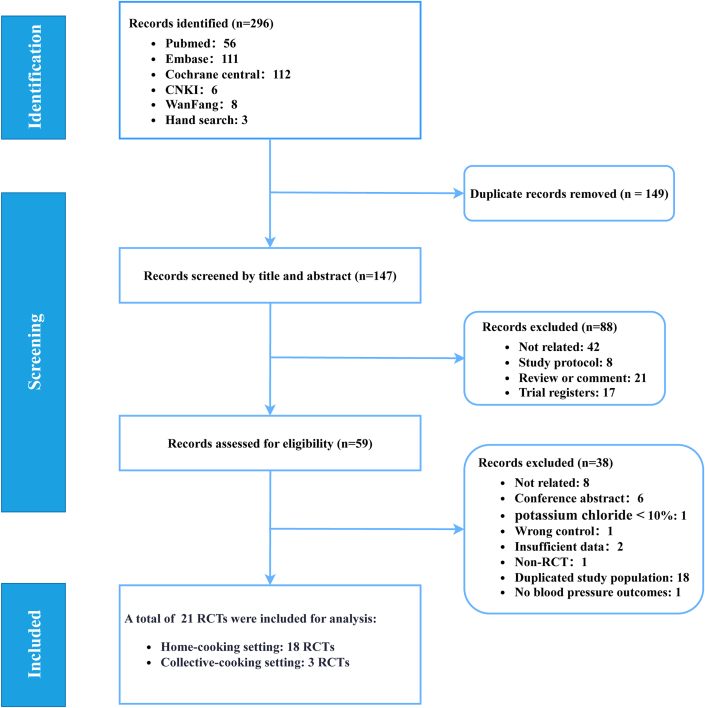
Flowchart of Literature Search and Selection The selection process for randomized controlled trials (RCTs) evaluating the effects of salt substitutes on blood pressure. A total of 296 records were identified. After removing duplicates, screening titles, and abstracts, and assessing full texts, 21 RCTs were included in the final analysis: 18 were conducted in home-cooking settings and 3 were in collective-cooking settings.

Twenty-one RCTs were eligible (Figure 1). Of these, 18 were individual randomized trials,^16^, ^17^, ^18^, ^19^, ^20^, ^21^, ^22^, ^23^, ^24^, ^25^, ^26^, ^27^, ^28^, ^29^, ^30^, ^31^, ^32^, ^33^ 2 were cluster randomized trials,^11^^,^^34^ and 1 was a stepped-wedge cluster randomized trial.^35^ Twelve RCTs (57.1%) were conducted in China.^11^^,^^21^, ^22^, ^23^, ^24^^,^^26^^,^^28^, ^29^, ^30^^,^^32^, ^33^, ^34^ Regarding the application setting, 18 RCTs (85.7%) were implemented in the setting of home-cooking,^16^^,^^18^, ^19^, ^20^, ^21^, ^22^, ^23^, ^24^^,^^26^, ^27^, ^28^, ^29^, ^30^, ^31^, ^32^, ^33^, ^34^, ^35^ whereas the remaining 3 (14.3%) were implemented in the setting of collective-cooking.^11^^,^^17^^,^^25^ Risk of bias assessment indicated low risk for 8 RCTs, some concerns for 10 RCTs, and high risk for 3 RCTs. In the home-cooking setting, 6 RCTs were judged to have low risk of bias, 9 had some concerns, and 3 were rated high risk of bias; in the collective-cooking setting, 2 RCTs were rated low risk of bias and 1 had some concerns (Supplemental Figure 1). Sample size ranged from 32 to 20,995 participants. The mean age of participants ranged from 20.9 to 71.0 years. The mean baseline SBP ranged from 113.1 to 176.9 mm Hg, and the mean baseline DBP ranged from 72.0 to 104.5 mm Hg. The proportion of male participants ranged from 14.3% to 76.3%. The proportion of potassium chloride ranged from 25% to 66%, with 1 study not reporting this information, and 14 (70%) studies using salt substitutes containing ≤30% potassium chloride. The intervention duration ranged from 1 month to 60 months with 61.9% (13 RCTs) being less than 12 months (Table 1).

**Table 1:** Characteristics of Included Studies

First Author, Year	Region	Design	Study Population	Application Setting for Cooking	Intervention Duration, mo	Sample Size	Male, %	Mean Age, y	Baseline mean SBP/DBP, mm Hg	% of KCI
Suppa et al,^16^ 1988	Italy	RCT	HTN	Home	1	322	62.7	47.4	147.2/95.4	25
Geleijnse et al,^17^ 1994	Netherlands	RCT	HTN	Collective	6	100	51.0	66.4	157.7/90.3	41
Omvik and Myking,^18^ 1995	Norway	RCT	HTN	Home	6	40	67.5	44.3	165.5/103.5	28
Gilleran et al,^19^ 1996	UK	RCT	HTN	Home	3	40	60.0	60.9	169.3/92.8	40
Pereira et al,^20^ 2005	Brazil	RCT	HTN	Home	3	22	14.3	48.3	138.3/92.9	50
CSSS-2,^21^ 2006	China	RCT	HTN	Home	12	220	63.2	57.4	156.7/90.4	25
			Mixed	Home	12	348	55.5	45.6	142.5/83.5	25
CSSS,^22^ 2007	China	RCT	Mixed	Home	12	608	44.1	60.0	159.0/93.0	25
Zhou et al,^23^ 2009	China	RCT	HTN	Home	6	126	42.9	66.6	158.7/83.0	30
			Normotensive	Home	6	122	46.7	66.7	124.4/74.4	30
Mu et al,^24^ 2009	China	RCT	HTN	Home	24	215	53.5	20.9	124.1/76.1	NA
Sarkkinenet al,^25^ 2011	Finland	RCT	Mixed	Collective	2	45	51.1	55.5	136.9/88.5	25
Zhao et al,^26^ 2014	China	RCT	HTN	Home	3	282	41.1	63.2	176.9/104.5	25
Barros et al,^27^ 2015	Brazil	RCT	HTN	Home	1	38	34.3	55.5	143.2/90.5	66
Zhou et al,^28^ 2016	China	RCT	Mixed	Home	36	462	49.4	46.4	151.7/90.2	25
Yang et al,^29^ 2018	China	RCT	HTN	Home	6	51	41.2	66.8	158.9/81.0	30
			HTN	Home	6	75	44.0	66.4	158.0/84.4	30
Bernabe-Ortiz et al,^35^ 2020	Peru	Stepped-wedge cRCT	Mixed	Home	4∼30	2,376	49.6	43.3	113.1/72.0	25
Li et al,^30^ 2021	China	RCT	Mixed	Home	2	516	50.0	59.2	127.8/81.6	30
Yu et al,^31^ 2021	India	RCT	HTN	Home	3	502	41.2	61.6	132.5/83.3	30
Neal et al,^34^ 2021	China	cRCT	Mixed	Home	60	20,995	50.5	65.4	154.0/89.2	25
Che et al,^32^ 2022	China	RCT	HTN	Home	12	322	40.4	62.6	134.3/77.8	32
Yuan et al,^11^ 2023	China	cRCT	Mixed	Collective	24	1,612	76.3	71.0	137.5/80.5	25
Zhang et al,^33^ 2023	China	RCT	HTN	Home	1	64	42.2	69.0	137.8/79.5	31
			HTN	Home	1	67	49.3	65.8	134.6/78.9	56

cRCT = cluster randomized controlled trial; DBP = diastolic blood pressure; HTN = hypertension; NA = not available; RCT = randomized controlled trial; SBP = systolic blood pressure.

### Overall effect of salt substitutes on blood pressure in all participants

**Figure 2:**
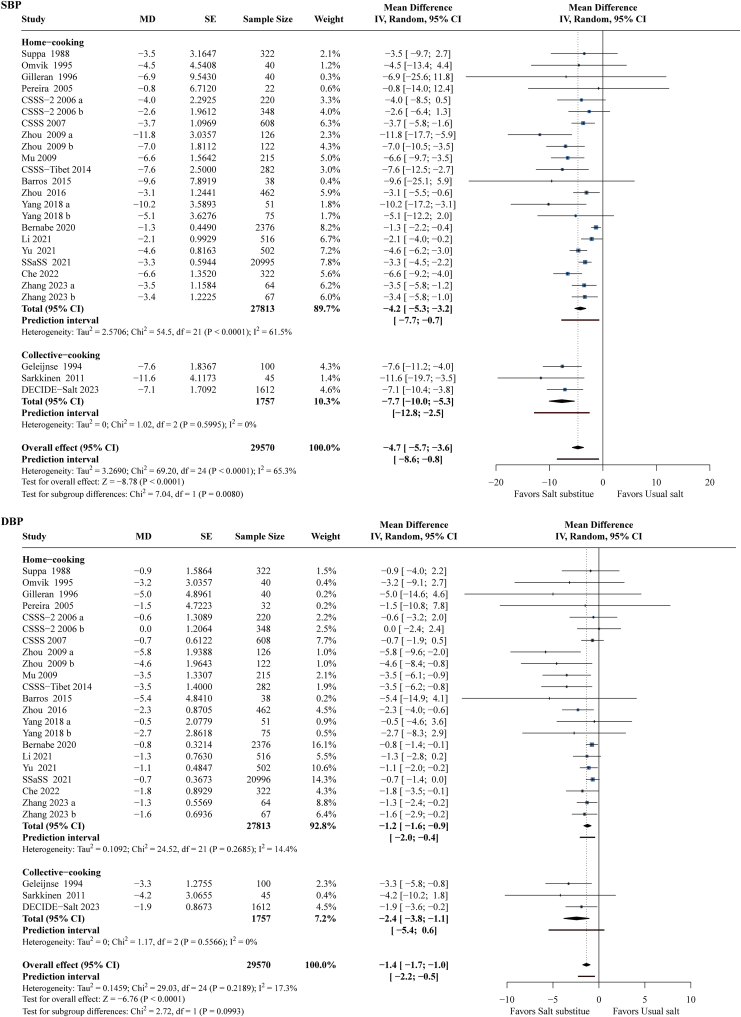
Effects of Salt Substitutes on Systolic and Diastolic Blood Pressure The squares represent the effect of salt substitutes on blood pressure in each RCT. The diamonds represent the blood pressure–lowering effect of salt substitutes within each application setting and overall, respectively. The horizontal lines represent the prediction interval. Random-effects meta-analysis with DerSimonian-Laird estimation was used to pool effect sizes. DBP = diastolic blood pressure; IV = inverse variance; MD = mean difference; SE = standard error; SBP = systolic blood pressure; other abbreviation as in Figure 1.

Meta-analysis of the 21 included RCTs demonstrated that, compared to usual salt, salt substitutes significantly reduced SBP (MD: –4.7 mm Hg; 95%CI: -5.7 to -3.6; I^2^=65.3%, high-certainty) and DBP (MD: –1.4 mm Hg; 95% CI: –1.7 to –1.0; I^2^ = 17.3%, high-certainty) (Figure 2, Supplemental Table 2).

### Effect of salt substitutes in different application settings

In the setting of home cooking (18 RCTs, 27,813 participants), salt substitutes reduced SBP (MD: –4.2 mm Hg; 95% CI: –5.3 to –3.2; I^2^ = 61.5%, high certainty) and DBP (MD: –1.2 mm Hg; 95% CI: –1.6 to –0.9; I^2^ = 14.4%, high certainty). In contrast, in the setting of collective cooking (3RCTs, 1,757 participants), SBP was reduced by a mean of 7.7 mm Hg (95% CI: –10.0 to –5.3; I^2^ = 0%, high certainty) and DBP by a mean of 2.4 mm Hg (95% CI: –3.8 to –1.1; I^2^ = 0%, high certainty). Subgroup meta-analysis indicated that the reduction in SBP was significantly greater in the collective-cooking setting compared to the home-cooking setting (*P* = 0.008) and the reduction in DBP was borderline significant (*P* = 0.099) (Figure 2, Supplemental Table 2).

### Sensitivity analyses

**Figure 3:**
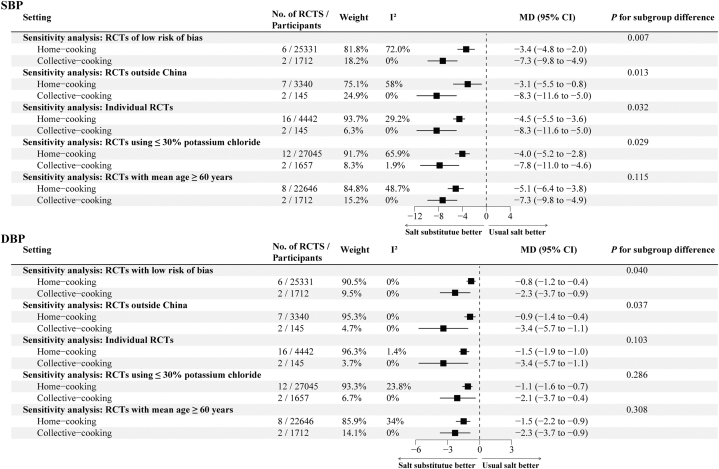
Results From Sensitivity Analyses Except for the Leave-One-Out Analysis The square represents the effect of salt substitutes on blood pressure in each application setting. MD and 95% CIs were pooled by meta-analysis using random-effects model. *P* values for difference were derived from Cochran’s Q test. Chi square statistics and corresponding *P* values for heterogeneity for each sensitivity analysis are provided in Supplemental Table 3. Abbreviations as in Figures 1 and 2.

Sensitivity analysis restricted to RCTs with low risk of bias showed that salt substitutes reduced SBP by 7.3 mm Hg (95% CI: −9.8 to −4.9) and DBP by 2.3 mm Hg (95% CI: −3.7 to −0.9) in the collective-cooking setting. These reductions were significantly greater than those observed in the home-cooking setting, where SBP and DBP were reduced by 3.4 mm Hg (95% CI: −4.8 to −2.0) and 0.8 mm Hg (95% CI: −1.2 to −0.4), respectively (Figure 3). In multivariable meta-regression, compared with the home-cooking setting, the collective-cooking setting was associated with an additional reduction of 3.5 mm Hg in SBP (95% CI: −6.4 to −0.7; *P* = 0.016) and 1.5 mm Hg in DBP (95% CI: −3.0 to −0.1; *P* = 0.037) after adjustment for baseline mean blood pressure, mean age, proportion of potassium chloride in salt substitutes, region, intervention duration, and risk of bias.

**Figure 4:**
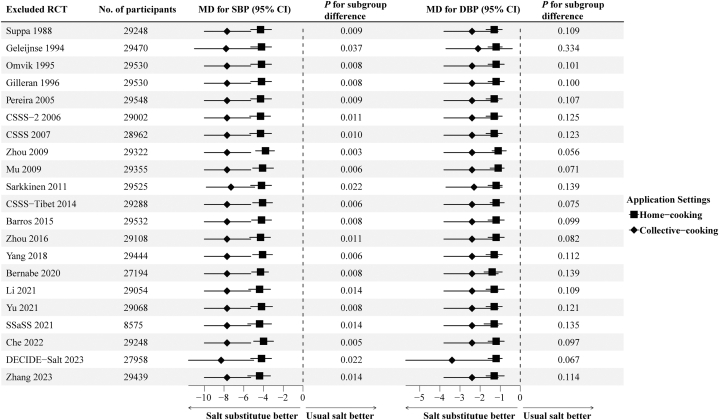
Results From the Leave-One-Out Sensitivity Analysis The squares and diamonds represent effects of salt substitutes on blood pressure in home-cooking and collective-cooking settings, respectively, based on leave-one-out analysis. MD and 95% CIs were pooled by meta-analysis using random-effects model. *P* for difference were estimated by Cochran’s Q test. Chi square statistics, I^2^, and corresponding *P* values for heterogeneity are provided in Supplemental Table 4. Abbreviations as in Figures 1 and 2.

Analyses restricted to individual RCTs, RCTs conducted outside China, RCTs using salt substitutes containing ≤30% potassium chloride, and RCTs with a mean age ≥60 years all confirmed similar results (Figure 3). Our leave-one-out analysis showed that no single study would have changed the above results significantly (Figure 4).

## Discussion

The present study systematically reviewed RCTs investigating the effect of potassium-enriched salt substitutes on blood pressure and performed meta-analyses to compare the effect between 2 specific application settings. We found that replacing usual salt with salt substitutes in a collective-cooking setting resulted in a greater reduction in blood pressure compared to a home-cooking setting (Central Illustration).

**Central Illustration:**
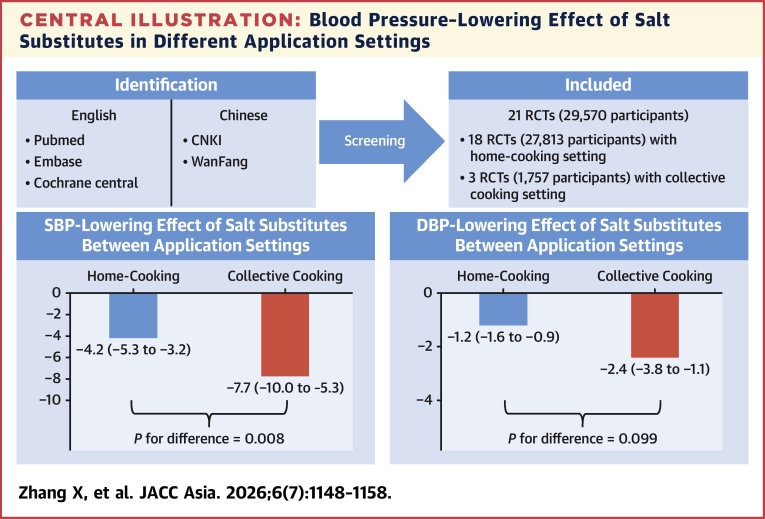
Blood Pressure–Lowering Effect of Salt Substitutes in Different Application Settings Evidence from 21 RCTs (29,570 participants). Of these, 18 were conducted in home-cooking settings and 3 in collective-cooking settings. Pooled analyses show larger reductions in SBP with salt substitutes in collective-cooking than in home-cooking settings. The reduction in DBP was borderline significant. DBP = diastolic blood pressure; RCT = randomized controlled trial; SBP = systolic blood pressure.

Previous systematic reviews have shown that replacing usual salt with salt substitutes can significantly reduce blood pressure.^10^^,^^36^ However, no studies have explored whether this effect varies across different application settings. The enhanced effect observed in a collective-cooking setting can be attributed to the unique dietary circumstances, where the quantity of salt or salt substitutes used in cooking or food preparation is regulated by a chef under managerial supervision, and consumers have minimal influence over their food choices and the amount of salt or salt substitute used. This controlled environment is expected to optimize the intervention's impact. Conversely, in a home-cooking setting, the use of salt substitutes is entirely at the discretion of the individual responsible for cooking. In this context, numerous factors can influence an individual's salt substitution behavior, such as their belief in the benefits of salt substitution, established seasoning habits, and family members' taste preferences. Studies have shown that dietary interventions implemented in controlled environments consistently achieve better adherence than those relying on individual behavior change.^37^

Our findings have significant implications for public health policy and practice. The additional reduction in SBP achieved through collective-cooking implementation could translate to substantial cardiovascular benefits at the population level. Such evidence can inform policy makers, health care providers, and administrators of residential institutions (eg, elderly care facilities, schools) to prioritize and promote the use of salt substitutes in such kind of setting. Given the growing proportion of meals consumed outside the home, particularly in urban areas and among working populations,^38^ targeting collective food service environments offers a strategic opportunity for population-wide sodium reduction. Policy interventions such as mandating or incentivizing the use of salt substitutes in institutional food services, workplace canteens, residential care facility cafeterias, and school canteens, could achieve broader public health impacts beyond those achievable through home cooking alone, as institutional procurement policies are a key lever for lowering population sodium intake.^39^ Practically, our findings support large-scale adoption of salt substitutes in collective-cooking settings, alongside their use in home cooking, as an effective strategy to enhance blood pressure control at the population level.

The estimated health gains at the population level could be substantial. A previous modeling study has reported that nationwide replacement of discretionary salt with potassium-enriched salt substitutes could avert approximately 461,000 cardiovascular deaths and 743,000 nonfatal cardiovascular events each year in China, based on a mean SBP reduction of 2.82 mm Hg achieved in the SSaSS (Salt Substitute and Stroke Study).^40^ Our study results indicate that if whole population strategies are implemented, the blood pressure-lowering effect as well as the projected number of averted fatal and nonfatal cardiovascular events would be doubled.

Safety and acceptability are key considerations for large-scale implementation of salt substitutes. The previous systematic review has well summarized both direct and indirect evidence on safety of using salt substitutes to replace usual salt in human beings from various types of studies including case reports, randomized trials, and cross-sectional and cohort studies. Although information on safety outcomes is limited, the use of salt substitutes, particularly with KCI <30%, should be considered safe in the general population except for those with impaired renal function, for whom serum potassium should be monitored.^41^ In the DECIDE-Salt trial, the only randomized trial that did not exclude individuals with high risk of hyperkalemia and was conducted among older people with a mean age of 70 years, including 6% participants with renal disease and 8% on potassium-raising medications, no clinical adverse outcomes were detected in association with the intervention, although the number of transit high serum potassium was increased.^11^ The SSaSS trial, where more than 10,000 households applied salt substitutes for 5 years, did not show an increased risk of clinical hyperkalemia or other clinical adverse events among adults at high cardiovascular risk.^34^

In terms of acceptability, studies have shown that when the potassium chloride content in low sodium salts was <30%, more than 80% of the subjects could not differentiate between usual salt and low sodium salt.^42^ In the SSaSS study with the home-cooking setting, 92% of participants in the intervention group continued using salt substitutes at the end of 5 years of intervention.^34^ In the China Salt Substitute Study, the acceptability of salt substitutes was comparable to usual salt.^43^ In the DECIDE-Salt trial with the collective-cooking setting, the compliance to salt substitute intervention was high; in contrast, the salt supply restriction intervention failed,^11^ indicating that salt substitution is much more acceptable than salt restriction.

### Study limitations

Our systematic review is based exclusively on data from RCTs and the results are confirmed by multiple sensitivity analyses, including those restricted to high-quality studies and using meta-regression model adjusting for multiple variables. Our findings are both robust and credible.

The study also has limitations. First, the number of RCTs with salt substitute use in collective-cooking settings is relatively small. However, the consistency in the intervention effects among studies is high, as indicated by the relatively narrow 95% CIs. Second, we did not systematically synthesize safety and acceptability outcomes; dedicated systematic reviews and meta-analyses focused on these aspects of salt substitutes are warranted. Third, 13 of 21 included studies judged having “some concerns” or “high risk” of bias; however, these trials together contributed only 8.5% of the total number of participants, indicating that the pooled estimates were mainly driven by studies with low risk of bias. Fourth, most of the included studies were conducted in China, although the sensitivity analyses restricted to studies conducted outside China showed similar effect estimates, supporting good external validity of our findings.

## Conclusions

Replacing usual salt with salt substitutes in a collective-cooking setting may lead to a greater reduction in blood pressure compared to the application in a home-cooking setting, suggesting that integrating salt substitution into public food procurement standards and routine catering practices could be an efficient strategy for population-wide blood pressure control. Public health policies and strategies should prioritize the full adoption of salt substitutes to maximize its health benefits.

## Funding Support and Author Disclosures

The study was supported and organized by the Health Risk Assessment and Control Committee, the Chinese Association of Preventive Medicine. Xianghui Zhang was supported by the Xinjiang Talent Development Fund (No. XJRC-2025-BTJY-YJ-GX-QNQZ-003), and Yifang Yuan was supported by the National Natural Science Foundation of China (No. 82404360). The funders had no role in the design of the study, data extraction, statistical analysis, interpretation of the results, writing of the report, or the decision to submit the article for publication. The authors have reported that they have no relationships relevant to the contents of this paper to disclose.
