# Supplementary material for: Blood Pressure Reduction Effects of Salt Substitutes in Different Application Settings A Meta-Analysis of Randomized Controlled Trials
Source: JACC Asia. 2026 Apr 1;6(7):1148–58. doi: 10.1016/j.jacasi.2026.02.014 (PMC13350103; doi:10.1016/j.jacasi.2026.02.014)
Supplement: Supplemental Material [file mmc1.docx]

**Supplemental Materials**

**Title: Blood Pressure Reduction Effects of Salt Substitutes in Different Application Settings: A Meta-Analysis of RCTs**

**Running title:** Salt Substitutes Reduced Blood Pressure by Application Settings

**Content**

**Search strategy…………………………………………………………………….…2**

**Table 1 Rationale for judging application setting …..……………………………..6**

**Table 2. GRADE summary of SBP and DBP in different application settings and overall……………………………………………………………….………………...8**

**Table 3. Pooled effects and heterogeneity statistics of sensitivity analyses……….9**

**Table 4. Pooled effects and heterogeneity statistics of leave-one-out sensitivity analyses……………………………………………………………….……………...10**

**Figure 1. Risk of bias assessment…………………...…………………………..….12**

**Figure 2: Baujat plot for overall blood pressure lowering effect of salt substitutes…………………………………………………………………………....13**

**Search strategy**

**PubMed**

**#1:**

((((((((((((((participant*) OR (subject*)) OR (male*)) OR (female*)) OR (men)) OR (man)) OR (women)) OR (woman)) OR (baby)) OR (babies)) OR (infant*)) OR (patient*)) OR (person)) OR (people)) OR (human*)

**#2:**

"salt substitute"[Title/Abstract] OR "salt substitutes"[Title/Abstract] OR "salt substitution"[Title/Abstract] OR "sodium substitute"[Title/Abstract] OR "sodium substitutes"[Title/Abstract] OR "sodium substitution"[Title/Abstract] OR "substituting sodium"[Title/Abstract] OR "sodium chloride substitute"[Title/Abstract] OR "sodium chloride substitutes"[Title/Abstract] OR "sodium chloride substitution"[Title/Abstract] OR "salt alternative"[Title/Abstract] OR "salt alternatives"[Title/Abstract] OR "sodium alternative"[Title/Abstract] OR "sodium alternatives"[Title/Abstract] OR "low sodium salt"[Title/Abstract] OR "low-sodium salt substitute"[Title/Abstract] OR "low-sodium salt substitutes"[Title/Abstract] OR "low-sodium salt substitution"[Title/Abstract] OR "sodium reduced salt"[Title/Abstract] OR "reduced-sodium salt"[Title/Abstract] OR "reduced sodium salt"[Title/Abstract] OR "sodium replacer"[Title/Abstract] OR "sodium replacers"[Title/Abstract] OR "sodium replacement"[Title/Abstract] OR "sodium-replacer"[Title/Abstract] OR "sodium-replacers"[Title/Abstract] OR "sodium-replacement"[Title/Abstract] OR "salt replacer"[Title/Abstract] OR "salt replacers"[Title/Abstract] OR "salt replacement"[Title/Abstract] OR "salt-replacer"[Title/Abstract] OR "salt-replacers"[Title/Abstract] OR "salt-replacement"[Title/Abstract] OR "sodium chloride replacer"[Title/Abstract] OR "sodium chloride replacers"[Title/Abstract] OR "sodium chloride replacement"[Title/Abstract] OR "potassium salt"[Title/Abstract] OR "KCl salt"[Title/Abstract] OR "potassium chloride salt"[Title/Abstract] OR "potassium-enriched salt"[Title/Abstract] OR "potassium enriched salt"[Title/Abstract] OR "potassium-based salt"[Title/Abstract] OR "potassium based salt"[Title/Abstract] OR "mineral salt"[Title/Abstract]

**#3:**

(("blood pressure"[Title/Abstract]) OR ("systolic blood pressure"[Title/Abstract])) OR ("diastolic blood pressure"[Title/Abstract])

**#4:**

("RCT"[Publication Type]) OR ("Randomized controlled trial"[Publication Type])

**#5:**

**#1 AND #2 AND #3 AND #4**

**Embase:**

**#1:**

'participant*' OR 'subject*' OR 'male*' OR 'female*' OR 'men'/exp OR 'men' OR 'man'/exp OR 'man' OR 'women'/exp OR 'women' OR 'woman'/exp OR 'woman' OR 'baby'/exp OR 'baby' OR 'babies' OR 'infant*' OR 'patient*' OR 'person' OR 'people' OR 'human*'

**#2:**

'salt substitute':ti,ab OR 'salt substitutes':ti,ab OR 'salt substitution':ti,ab OR 'sodium substitute':ti,ab OR 'sodium substitutes':ti,ab OR 'sodium substitution':ti,ab OR 'substituting sodium':ti,ab OR 'sodium chloride substitute':ti,ab OR 'sodium chloride substitutes':ti,ab OR 'sodium chloride substitution':ti,ab OR 'salt alternative':ti,ab OR 'salt alternatives':ti,ab OR 'sodium alternative':ti,ab OR 'sodium alternatives':ti,ab OR 'low sodium salt':ti,ab OR 'low-sodium salt substitute':ti,ab OR 'low-sodium salt substitutes':ti,ab OR 'low-sodium salt substitution':ti,ab OR 'sodium reduced salt':ti,ab OR 'reduced-sodium salt':ti,ab OR 'reduced sodium salt':ti,ab OR 'sodium replacer':ti,ab OR 'sodium replacers':ti,ab OR 'sodium replacement':ti,ab OR 'sodium-replacer':ti,ab OR 'sodium-replacers':ti,ab OR 'sodium-replacement':ti,ab OR 'salt replacer':ti,ab OR 'salt replacers':ti,ab OR 'salt replacement':ti,ab OR 'salt-replacer':ti,ab OR 'salt-replacers':ti,ab OR 'salt-replacement':ti,ab OR 'sodium chloride replacer':ti OR 'sodium chloride replacers':ti,ab OR 'sodium chloride replacement':ti,ab OR 'potassium salt':ti,ab OR 'kcl salt':ti,ab OR 'potassium chloride salt':ti,ab OR 'potassium-enriched salt':ti,ab OR 'potassium enriched salt':ti,ab OR 'potassium-based salt':ti,ab OR 'potassium based salt':ti,ab OR 'mineral salt'

**#3:**

'blood pressure':ti,ab OR 'systolic blood pressure':ti,ab OR 'diastolic blood pressure':ti,ab

**#4:**

'randomized controlled trial'

**#5:**

#1 AND #2 AND #3 AND #4

**Cochrane CENTRAL**

**#1:**

("participant*" OR "subject*" OR "male*" OR "female*" OR "men" OR "man" OR "women" OR "woman" OR "baby" OR "babies" OR "infant*" OR "patient*" OR "person" OR "people" OR "human*")

**#2:**

("salt substitute" OR "salt substitutes" OR "salt substitution" OR "sodium substitute" OR "sodium substitutes" OR "sodium substitution" OR "substituting sodium" OR "sodium chloride substitute" OR "sodium chloride substitutes" OR "sodium chloride substitution" OR "salt alternative" OR "salt alternatives" OR "sodium alternative" OR "sodium alternatives" OR "low sodium salt" OR "low-sodium salt substitute" OR "low-sodium salt substitutes" OR "low-sodium salt substitution" OR "sodium reduced salt" OR "reduced-sodium salt" OR "reduced sodium salt" OR "sodium replacer" OR "sodium replacers" OR "sodium replacement" OR "sodium-replacer" OR "sodium-replacers" OR "sodium-replacement" OR "salt replacer" OR "salt replacers" OR "salt replacement" OR "salt-replacer" OR "salt-replacers" OR "salt-replacement" OR "sodium chloride replacer" OR "sodium chloride replacers" OR "sodium chloride replacement" OR "potassium salt" OR "KCl salt" OR "potassium chloride salt" OR "potassium-enriched salt" OR "potassium enriched salt" OR "potassium-based salt" OR "potassium based salt" OR "mineral salt"):ti,ab,kw

**#3:**

(“blood pressure” OR “systolic blood pressure” OR “diastolic blood pressure”):ti,ab,kw

**#4:**

#1 AND #2 AND #3 in Trials

**CNKI**

(主题:人+男性+女性+人群+婴儿+病人+儿童+新生儿+患者)AND(主题:低钠盐 +代盐+富钾盐+富钾低钠盐+富钾替代盐)AND(主题:血压+收缩压+舒张压)AND(主题:随机对照试验)

**WanFang**

主题:(人 or 男性 or 女性 or 人群 or 婴儿 or 病人 or 儿童 or 新生儿 or 患者) and 主题:(低钠盐 or 代盐 or 富钾盐 or 富钾低钠盐 or 富钾替代盐) and 主题:(血压 or 收缩压 or 舒张压) and 主题:(随机对照试验)

**Table 1. Rationale for judging application setting**

| **Study, year** | **Country** | **Adjudication rationale** | **Application Setting** |
| --- | --- | --- | --- |
| Suppa 1988^12^ | Italy | Both salts were given at table, in double-blind conditions over a period of 4 weeks | Home-cooking |
| Geleijnse 1994^13^ | Netherlands | Trial foods included bread, cheese, luncheon meats, canned and instant soups, and smoked sausage. | Collective-cooking |
| Omvik 1995^14^ | Norway | The patients were asked to remove all the ordinary household salt and replace it with the salt from the Clinic. | Home-cooking |
| Gilleran 1996^15^ | UK | Unlabelled packs of one container of seltin (500 gm) and two salt cellar containers (90 gm) of Seltin or the equivalent packs and cellars of ordinary salt, were given to each patient for a monthly supply. At each monthly and subsequent three monthly visits, the unlabelled packs and cellars were returned and the amount recorded. | Home-cooking |
| Pereira 2005^16^ | Brazil | assurance that the meals were all prepared at home and taken to work. | Home-cooking |
| CSSS-2 2006^17^ | China | This study provides sufficient intervention salt to all participating families | Home-cooking |
| CSSS 2007^18^ | China | …salt substitute/salt available to each randomized participant to cover all cooking, pickling and other uses within the household. | Home-cooking |
| Zhou 2009^19^ | China | A total of 3 kg a month CISalt or NSalt was provided to each participant’s family to cover all cooking and other uses | Home-cooking |
| Mu 2009^20^ | China | …we supplied the families with salt to which potassium chloride and calcium chloride had been added. Our researchers visited to each family kitchen to assess the amount of salt consumed every month. | Home-cooking |
| Sarkkinen 2011^21^ | Finland | The main food sources of salt were either salted as normal or with Smart Salt®. Test foods were industrially processed main dishes (casseroles, soups, pastas, pizza and minced meat dishes), bread (70% rye bread and 30% multigrain), frankfurters sausage/cold cuts and Edam cheese. Salt used for cooking and baking as well as table salt was either regular salt or Smart Salt®, dependent on group. The use of products containing bioactive peptides (like Evolus®), salts other than the test salts, licorice (Radix glycyrrhizae), ammonium chloride products and any food supplements that might affect BP were also prohibited. | Collective-cooking |
| CSSS-Tibet 2014^22^ | China | The study salt was distributed free of charge to every household with sufficient amount to cover three months’ consumption for the whole household. | Home-cooking |

**Table 1-Continued**

| **Study, year** | **Country** | **Adjudication rationale** | **Application**  **Setting** |
| --- | --- | --- | --- |
| Barros 2015^23^ | Brazil | Every patient received 28 small plastic bags containing the daily amount of salt… | Home-cooking |
| Zhou 2016^24^ | China | Participants were asked to prepare all foods using their assigned study salt during the study period. | Home-cooking |
| Yang 2018^25^ | China | 1. This study…conducted in 10 communities in the rural Hedong District, Tianjin, China.  2. …the following inclusion criteria were applied: patients who ate no more than one meal per week outside of their home, … | Home-cooking |
| Bernabe 2020^31^ | Peru | Thus, common salt (NaCl) used in the enrolled households was retrieved and replaced, free of charge, with a salt substitute, … | Home-cooking |
| Li 2021^26^ | China | The family allocated to the intervention group received free salt substitute (70% sodium chloride and 30% potassium chloride). Sufficient salt substitute was provided to the entire family for the use of two months. | Home-cooking |
| Yu 2021^27^ | India | Participants were randomly assigned to receive either regular salt (100% sodium chloride) or the salt substitute (70% sodium chloride/30% potassium chloride blend), and advised to replace all home salt use. | Home-cooking |
| SSaSS 2021^30^ | China | Participants in the intervention villages were provided reduced sodium salt substitute free-of-charge as a replacement for regular salt. | Home-cooking |
| Che 2022^28^ | China | The participants were asked to replace the salt they used for cooking with the salt provided by the study. | Home-cooking |
| DECIDE-Salt 2023^10^ | China | Facilities assigned to the salt substitute group received salt substitute to replace usual salt. Facilities in the control group received usual salt, 100% sodium chloride, from the same company. Responsible staff were trained to store the salt in a locked room and supply it to the kitchen on a planned schedule, … | Collective-cooking |
| Zhang 2023^29^ | China | The test salt was used throughout the trial as a substitute for the participants’ household salt. | Home-cooking |

**Table 2. GRADE summary of SBP and DBP in different application settings and overall**

| **Outcomes** | **Effect** | **Certainty** | **Certainty assessment** | | | | | |  | **№ of participants** | |
| --- | --- | --- | --- | --- | --- | --- | --- | --- | --- | --- | --- |
|  |  |  | **№ of RCTs** | **Risk of bias** | **Inconsistency** | **Indirectness** | **Imprecision** | **Publication bias** |  | **Salt substitutes** | **Usual salt** |
| **Home-cooking** |  |  |  |  |  |  |  |  |  |  |  |
| SBP | -4.2 (-5.3 to -3.2) | ⨁⨁⨁⨁ High | 18 | not serious | not serious | not serious | not serious | none |  | 15083 | 12730 |
| DBP | -1.2 (-1.6 to -0.9) | ⨁⨁⨁⨁ High | 18 | not serious | not serious | not serious | not serious | none |  | 15083 | 12730 |
| **Collective-cooking** |  |  |  |  |  |  |  |  |  |  |  |
| SBP | -7.7 (-10.0 to -5.3) | ⨁⨁⨁⨁ High | 3 | not serious | not serious | not serious | not serious | none |  | 878 | 879 |
| DBP | -2.4 (-3.8 to -1.1) | ⨁⨁⨁⨁ High | 3 | not serious | not serious | not serious | not serious | none |  | 878 | 879 |
| **Overall** |  |  |  |  |  |  |  |  |  |  |  |
| SBP | -4.7 (-5.7 to -3.6) | ⨁⨁⨁⨁ High | 21 | not serious | not serious | not serious | not serious | none |  | 15961 | 13609 |
| DBP | -1.4 (-1.7 to -1.0) | ⨁⨁⨁⨁ High | 21 | not serious | not serious | not serious | not serious | none |  | 15961 | 13609 |

**Table 3. Pooled effects and heterogeneity statistics of sensitivity analyses**

| **Setting** | **SBP** | | | |  | **DBP** | | | |
| --- | --- | --- | --- | --- | --- | --- | --- | --- | --- |
|  | **MD (95% CI)** | **I^2^** | **Chi^2^** | ***P*** |  | **MD (95% CI)** | **I2** | **Chi2** | ***P*** |
| **Sensitivity analysis: RCTs of low risk of bias** |  |  |  |  |  |  |  |  |  |
| Home-cooking | -3.4 (-4.8, -2.0) | 72.0% | 21.42 | 0.002 |  | -0.8 (-1.2, -0.4) | 0.0% | 4.74 | 0.578 |
| Collective-cooking | -7.3 (-9.8, -4.9) | 0.0% | 0.04 | 0.842 |  | -2.3 (-3.7, -0.9) | 0.0% | 0.82 | 0.364 |
| **Sensitivity analysis: RCTs outside China** |  |  |  |  |  |  |  |  |  |
| Home-cooking | -3.1 (-5.5, -0.8) | 58.0% | 14.3 | 0.027 |  | -0.9 (-1.4, -0.4) | 0.0% | 2.51 | 0.867 |
| Collective-cooking | -8.3 (-11.6, -5.0) | 0.0% | 0.79 | 0.375 |  | -3.4 (-5.7, -1.1) | 0.0% | 0.07 | 0.786 |
| **Sensitivity analysis: Individual RCTs** |  |  |  |  |  |  |  |  |  |
| Home-cooking | -4.5 (-5.5, -3.6) | 29.2% | 26.83 | 0.109 |  | -1.5 (-1.9, -1.0) | 1.4% | 19.28 | 0.439 |
| Collective-cooking | -8.3 (-11.6, -5.0) | 0.0% | 0.79 | 0.375 |  | -3.4 (-5.7, -1.1) | 0.0% | 0.07 | 0.786 |
| **Sensitivity analysis: RCTs using <= 30% potassium chloride** |  |  |  |  |  |  |  |  |  |
| Home-cooking | -4.0 (-5.2, -2.8) | 65.9% | 41.1 | <0.001 | | -1.1 (-1.6, -0.7) | 23.8% | 18.38 | 0.19 |
| Collective-cooking | -7.8 (-11.0, -4.6) | 1.9% | 1.02 | 0.313 |  | -2.1 (-3.7, -0.4) | 0.0% | 0.52 | 0.47 |
| **Sensitivity analysis: RCTs with mean age >= 60 years** |  |  |  |  |  |  |  |  |  |
| Home-cooking | -5.1 (-6.4, -3.8) | 48.7% | 19.5 | 0.034 |  | -1.5 (-2.2, -0.9) | 34.0% | 15.15 | 0.127 |
| Collective-cooking | -7.3 (-9.8, -4.9) | 0.0% | 0.04 | 0.842 |  | -2.3 (-3.7, -0.9) | 0.0% | 0.82 | 0.364 |

**Table 4. Pooled effects and heterogeneity statistics of leave-one-out sensitivity analyses**

| **Excluded RCT** | **SBP** | | | |  | **DBP** | | | |
| --- | --- | --- | --- | --- | --- | --- | --- | --- | --- |
|  | **MD (95% CI)** | **I^2^** | **Chi^2^** | ***P*** |  | **MD (95% CI)** | **I^2^** | **Chi^2^** | ***P*** |
| **Suppa 1988** |  |  |  |  |  |  |  |  |  |
| Home-cooking | -4.3 (-5.3, -3.2) | 63.3% | 54.49 | <0.001 |  | -1.3 (-1.7, -0.9) | 18.4% | 24.5 | 0.221 |
| Collective-cooking | -7.7 (-10.0, -5.3) | 0.0% | 1.02 | 0.6 |  | -2.4 (-3.8, -1.1) | 0.0% | 1.17 | 0.557 |
| **Geleijnse 1994** |  |  |  |  |  |  |  |  |  |
| Home-cooking | -4.2 (-5.3, -3.2) | 61.5% | 54.5 | <0.001 |  | -1.2 (-1.6, -0.9) | 14.4% | 24.52 | 0.269 |
| Collective-cooking | -7.8 (-11.0, -4.6) | 1.9% | 1.02 | 0.313 |  | -2.1 (-3.7, -0.4) | 0.0% | 0.52 | 0.47 |
| **Omvik 1995** |  |  |  |  |  |  |  |  |  |
| Home-cooking | -4.2 (-5.3, -3.2) | 63.2% | 54.42 | <0.001 |  | -1.2 (-1.6, -0.8) | 16.9% | 24.05 | 0.24 |
| Collective-cooking | -7.7 (-10.0, -5.3) | 0.0% | 1.02 | 0.56 |  | -2.4 (-3.8, -1.1) | 0.0% | 1.17 | 0.557 |
| **Gilleran 1996** |  |  |  |  |  |  |  |  |  |
| Home-cooking | -4.2 (-5.3, -3.2) | 63.2% | 54.35 | <0.001 |  | -1.2 (-1.6, -0.8) | 16.3% | 23.89 | 0.247 |
| Collective-cooking | -7.7 (-10.0, -5.3) | 0.0% | 1.02 | 0.56 |  | -2.4 (-3.8, -1.1) | 0.0% | 1.17 | 0.557 |
| **Pereira 2005** |  |  |  |  |  |  |  |  |  |
| Home-cooking | -4.3 (-5.3, -3.2) | 63.2% | 54.38 | <0.001 |  | -1.3 (-1.7, -0.9) | 18.4% | 24.5 | 0.221 |
| Collective-cooking | -7.7 (-10.0, -5.3) | 0.0% | 1.02 | 0.6 |  | -2.4 (-3.8, -1.1) | 0.0% | 1.17 | 0.557 |
| **CSSS-2 2006** |  |  |  |  |  |  |  |  |  |
| Home-cooking | -4.3 (-5.4, -3.3) | 65.0% | 54.27 | <0.001 |  | -1.3 (-1.7, -0.9) | 18.9% | 23.43 | 0.219 |
| Collective-cooking | -7.7 (-10.0, -5.3) | 0.0% | 1.02 | 0.6 |  | -2.4 (-3.8, -1.1) | 0.0% | 1.17 | 0.557 |
| **CSSS 2007** |  |  |  |  |  |  |  |  |  |
| Home-cooking | -4.3 (-5.4, -3.2) | 63.1% | 54.26 | <0.001 |  | -1.3 (-1.7, -0.9) | 16.6% | 23.99 | 0.243 |
| Collective-cooking | -7.7 (-10.0, -5.3) | 0.0% | 1.02 | 0.6 |  | -2.4 (-3.8, -1.1) | 0.0% | 1.17 | 0.557 |
| **Zhou 2009** |  |  |  |  |  |  |  |  |  |
| Home-cooking | -3.8 (-4.8, -2.9) | 54.4% | 41.65 | 0.002 |  | -1.1 (-1.4, -0.7) | 0.0% | 15.47 | 0.693 |
| Collective-cooking | -7.7 (-10.0, -5.3) | 0.0% | 1.02 | 0.6 |  | -2.4 (-3.8, -1.1) | 0.0% | 1.17 | 0.557 |
| **Mu 2009** |  |  |  |  |  |  |  |  |  |
| Home-cooking | -4.1 (-5.1, -3.0) | 59.6% | 49.56 | <0.001 |  | -1.1 (-1.5, -0.8) | 6.1% | 21.29 | 0.38 |
| Collective-cooking | -7.7 (-10.0, -5.3) | 0.0% | 1.02 | 0.6 |  | -2.4 (-3.8, -1.1) | 0.0% | 1.17 | 0.557 |
| **Sarkkinen 2011** |  |  |  |  |  |  |  |  |  |
| Home-cooking | -4.2 (-5.3, -3.2) | 61.5% | 54.5 | <0.001 |  | -1.2 (-1.6, -0.9) | 14.4% | 24.52 | 0.269 |
| Collective-cooking | -7.3 (-9.8, -4.9) | 0.0% | 0.04 | 0.842 |  | -2.3 (-3.7, -0.9) | 0.0% | 0.82 | 0.364 |
| **CSSS-Tibet 2014** |  |  |  |  |  |  |  |  |  |
| Home-cooking | -4.1 (-5.1, -3.0) | 61.0% | 51.33 | <0.001 |  | -1.2 (-1.5, -0.8) | 7.5% | 21.61 | 0.362 |
| Collective-cooking | -7.7 (-10.0, -5.3) | 0.0% | 1.02 | 0.6 |  | -2.4 (-3.8, -1.1) | 0.0% | 1.17 | 0.557 |
| **Barros 2015** |  |  |  |  |  |  |  |  |  |
| Home-cooking | -4.2 (-5.2, -3.2) | 62.9% | 53.84 | <0.001 |  | -1.2 (-1.6, -0.8) | 15.8% | 23.74 | 0.254 |
| Collective-cooking | -7.7 (-10.0, -5.3) | 0.0% | 1.02 | 0.6 |  | -2.4 (-3.8, -1.1) | 0.0% | 1.17 | 0.557 |
| **Zhou 2016** |  |  |  |  |  |  |  |  |  |
| Home-cooking | -4.3 (-5.4, -3.3) | 63.3% | 54.5 | <0.001 |  | -1.2 (-1.6, -0.8) | 11.9% | 22.71 | 0.304 |
| Collective-cooking | -7.7 (-10.0, -5.3) | 0.0% | 1.02 | 0.6 |  | -2.4 (-3.8, -1.1) | 0.0% | 1.17 | 0.557 |
| **Yang 2018** |  |  |  |  |  |  |  |  |  |
| Home-cooking | -4.1 (-5.1, -3.1) | 62.3% | 50.37 | <0.001 |  | -1.3 (-1.7, -0.9) | 21.3% | 24.13 | 0.191 |
| Collective-cooking | -7.7 (-10.0, -5.3) | 0.0% | 1.02 | 0.6 |  | -2.4 (-3.8, -1.1) | 0.0% | 1.17 | 0.557 |
| **Bernabe 2020** |  |  |  |  |  |  |  |  |  |
| Home-cooking | -4.3 (-5.2, -3.5) | 30.5% | 28.78 | 0.092 |  | -1.4 (-1.8, -0.9) | 12.1% | 22.75 | 0.301 |
| Collective-cooking | -7.7 (-10.0, -5.3) | 0.0% | 1.02 | 0.6 |  | -2.4 (-3.8, -1.1) | 0.0% | 1.17 | 0.557 |
| **Li 2021** |  |  |  |  |  |  |  |  |  |
| Home-cooking | -4.4 (-5.5, -3.3) | 62.5% | 53.27 | <0.001 |  | -1.3 (-1.7, -0.8) | 18.3% | 24.47 | 0.223 |
| Collective-cooking | -7.7 (-10.0, -5.3) | 0.0% | 1.02 | 0.6 |  | -2.4 (-3.8, -1.1) | 0.0% | 1.17 | 0.557 |
| **Yu 2021** |  |  |  |  |  |  |  |  |  |
| Home-cooking | -4.2 (-5.3, -3.1) | 60.9% | 51.1 | <0.001 |  | -1.3 (-1.7, -0.9) | 18.4% | 24.52 | 0.221 |
| Collective-cooking | -7.7 (-10.0, -5.3) | 0.0% | 1.02 | 0.6 |  | -2.4 (-3.8, -1.1) | 0.0% | 1.17 | 0.557 |
| **SSaSS 2021** |  |  |  |  |  |  |  |  |  |
| Home-cooking | -4.4 (-5.6, -3.2) | 63.2% | 54.4 | <0.001 |  | -1.3 (-1.8, -0.9) | 11.4% | 22.58 | 0.31 |
| Collective-cooking | -7.7 (-10.0, -5.3) | 0.0% | 1.02 | 0.6 |  | -2.4 (-3.8, -1.1) | 0.0% | 1.17 | 0.557 |

**Table 4. Continue**

| **Excluded RCT** | **SBP** | | | |  | **DBP** | | | |
| --- | --- | --- | --- | --- | --- | --- | --- | --- | --- |
|  | **MD (95% CI)** | **I^2^** | **Chi^2^** | **P** |  | **MD (95% CI)** | **I^2^** | **Chi^2^** | **P** |
| **Che 2022** |  |  |  |  |  |  |  |  |  |
| Home-cooking | -4.0 (-5.0, -3.0) | 58.2% | 47.8 | <0.001 |  | -1.2 (-1.6, -0.8) | 16.4% | 23.93 | 0.245 |
| Collective-cooking | -7.7 (-10.0, -5.3) | 0.0% | 1.02 | 0.6 |  | -2.4 (-3.8, -1.1) | 0.0% | 1.17 | 0.557 |
| **DECIDE-Salt 2023** |  |  |  |  |  |  |  |  |  |
| Home-cooking | -4.2 (-5.3, -3.2) | 61.5% | 54.5 | <0.001 |  | -1.2 (-1.6, -0.9) | 14.4% | 24.52 | 0.269 |
| Collective-cooking | -8.3 (-11.6, -5.0) | 0.0% | 0.79 | 0.375 |  | -3.4 (-5.7, -1.1) | 0.0% | 0.07 | 0.786 |
| **Zhang 2023** |  |  |  |  |  |  |  |  |  |
| Home-cooking | -4.4 (-5.6, -3.3) | 65.1% | 54.37 | <0.001 |  | -1.3 (-1.7, -0.8) | 20.8% | 23.99 | 0.197 |
| Collective-cooking | -7.7 (-10.0, -5.3) | 0.0% | 1.02 | 0.6 |  | -2.4 (-3.8, -1.1) | 0.0% | 1.17 | 0.557 |


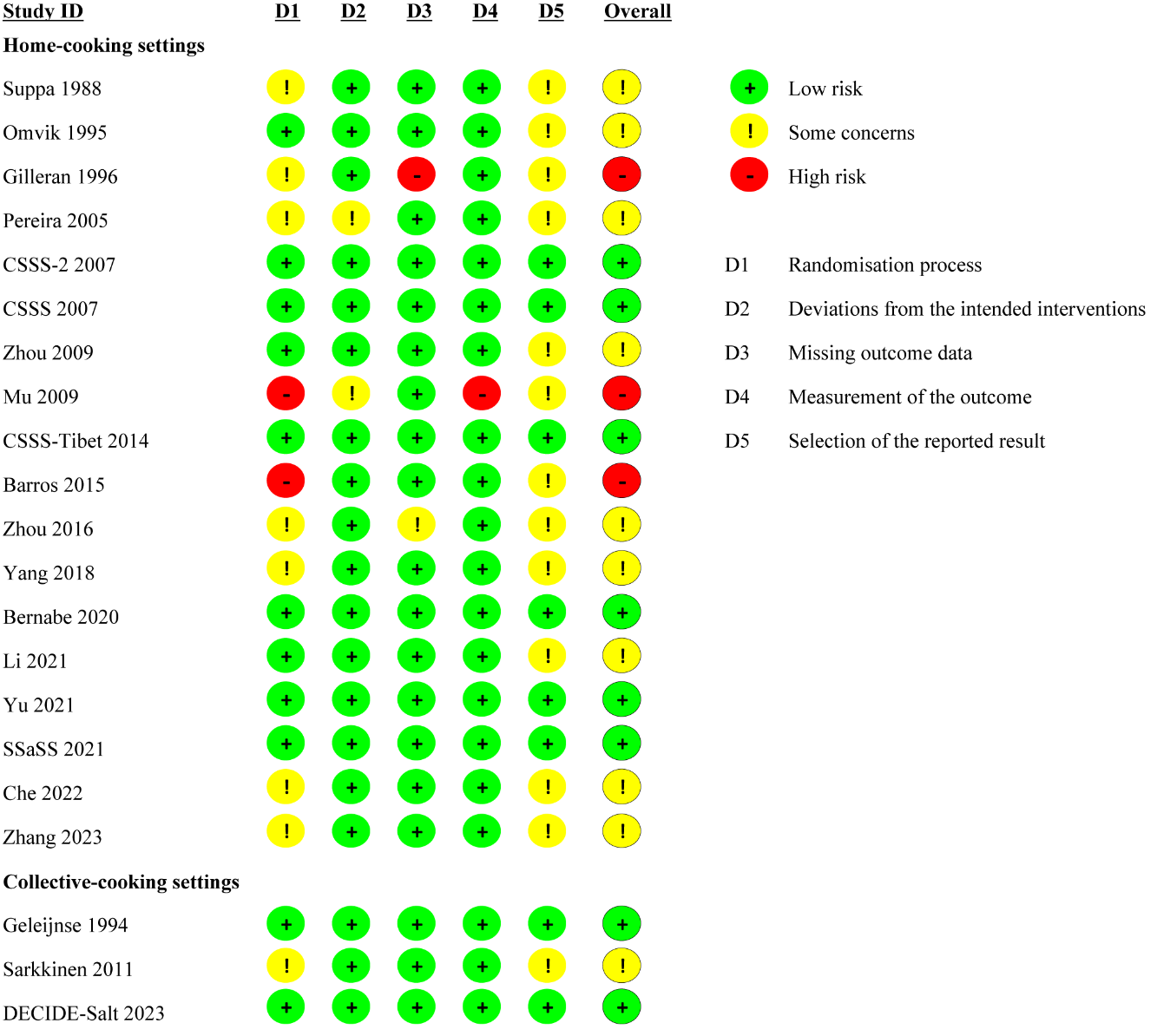


**Figure 1. Risk of bias assessment**


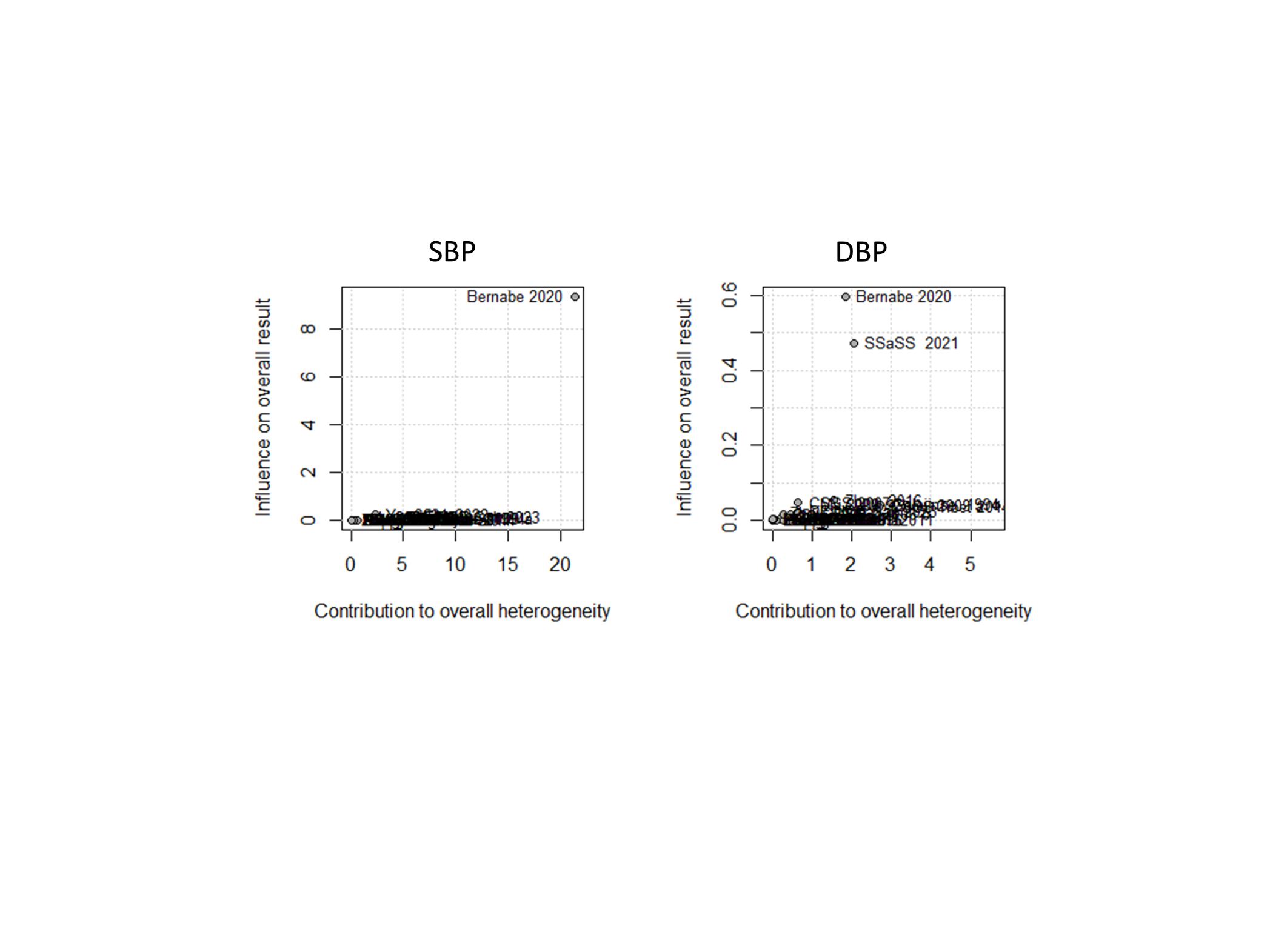


**Figure 2: Baujat plot for overall blood pressure lowering effect of salt substitutes**
